# What Is a Digital Twin? Experimental Design for a Data-Centric Machine Learning Perspective in Health

**DOI:** 10.3390/ijms232113149

**Published:** 2022-10-29

**Authors:** Frank Emmert-Streib, Olli Yli-Harja

**Affiliations:** 1 Predictive Society and Data Analytics Lab, Faculty of Information Technology and Communication Sciences, Tampere University, 33100 Tampere, Finland; 2 Computational Systems Biology, Faculty of Medicine and Health Technology, Tampere University, 33720 Tampere, Finland; 3 Institute for Systems Biology, Seattle, WA 98195, USA

**Keywords:** digital twin, data science, machine learning, experimental design, genomics, personalized medicine

## Abstract

The idea of a digital twin has recently gained widespread attention. While, so far, it has been used predominantly for problems in engineering and manufacturing, it is believed that a digital twin also holds great promise for applications in medicine and health. However, a problem that severely hampers progress in these fields is the lack of a solid definition of the concept behind a digital twin that would be directly amenable for such big data-driven fields requiring a statistical data analysis. In this paper, we address this problem. We will see that the term ’digital twin’, as used in the literature, is like a Matryoshka doll. For this reason, we unstack the concept via a data-centric machine learning perspective, allowing us to define its main components. As a consequence, we suggest to use the term Digital Twin System instead of digital twin because this highlights its complex interconnected substructure. In addition, we address ethical concerns that result from treatment suggestions for patients based on simulated data and a possible lack of explainability of the underling models.

## 1. Introduction

The construct of a digital twin is a fascinating idea. It is widely credited to Michael Grieves, who described it informally in 2002 for the formation of a product lifecycle management (PLM); however, without using its current name. Later, he provided a description of a digital twin by stating [1]:

The digital twin is a set of virtual information constructs that fully describes a potential or actual physical manufactured product from the micro atomic level to the macro geometrical level.

For completeness, we would like to remark that there are mentions of a digital twin in the literature earlier than 2002. For instance, in 1994, a realistic model of arteries was called a digital twin [2]. Even earlier, David Gelernter described in his book in 1991 a software model of reality, which he called a ‘mirror world’ instead of a digital twin, as a complete mirror image of reality that is constantly updated by sensor information [3].

In industry, the idea of a digital twin was met with great interest and notable early adoptions thereof were in astronautics and aerospace by NASA [4] and in manufacturing [5,6,7,8]. Since then, many authors have given a similar characterization of a digital twin, e.g., [9]:

A digital twin is a virtual representation that serves as the real-time digital counterpart of a physical object or process and addresses every instance for its total life cycle.

As a side note, we think it is interesting to remark that, recently, the idea of a digital twin is also explored in climate science. There, a digital twin of the structure and dynamics of the climate system of the Earth is currently developed to study different aspects of climate change and climate forecasts [10,11].

In contrast to those fields, much later, biology, medicine and health started to embrace the idea of a digital twin. In [12], a reason for this delay has been attributed to the increased difficulty in developing a ‘biological’ digital twin for such fields compared to engineered devices forming a ‘mechanical’ digital twin, e.g., a jet engine. In addition, ideally, a biological twin would require multiscale-modeling [13,14,15,16] on the subcellular, cellular, multicellular, tissue, organ and organism level, which is very challenging given our current knowledge about these levels.

Maybe the best application example to this day that has been clinically tested is the artificial pancreas model used to treat patients with type I diabetes [17]. The artificial pancreas is a mathematical model that simulates the glucose metabolism for a target patient. Specifically, the simulation model performs a closed-loop control by receiving real-time blood glucose levels via a sensor from the patient, which is used to predict the required insulin level. In case of a deviation, a pump attached to the patient injects the appropriate dose of insulin [18]. While this particular result is certainly very impressive, it focuses only on a single level of the human body. This is in contrast to [19], where a proof-of concept example has been studied that integrates information at the organ, tissue and cellular level. Unfortunately, the presented results of the study are only for simulated data. It is interesting to note that, so far, there is no example in the literature of a biological digital twin on the subcellular (genomics) level based on real data. Instead, there are a number of survey or perspective papers about a biological digital twin focusing either on the illustration of the underlying idea or the description of prospects thereof. For instance, disease-specific prospects can be found in [20,21] and general discussions can be found in [22,23,24,25].

Despite all of this progress in different fields, in our opinion, there is a conceptual problem with a digital twin that has not been properly addressed so far. Specifically, the term ‘digital twin’ is used throughout the literature in a cluttered, unprecise and convoluted way that hides rather than explicates design principles. In this paper, we address this issue by presenting a data-centric machine learning perspective on a digital twin. This will reveal the substructure behind a ‘digital twin’ and enables, in a natural way, an experimental design amenable for a statistical data analysis by utilizing methods from machine learning, artificial intelligence and statistics, which could be used for personalized and precision medicine [26,27,28]. Despite the general character of our discussion in the following, we focus on a biological digital twin (for problems in medicine and health), but the provided definitions should be extendable to the inanimate nature applicable to a mechanical digital twin for engineering.

## 2. What Is a Biological Digital Twin?

We start our discussion by specifying the concept of a biological digital twin. In order to introduce this concept in detail, we compare it with a biological twin. For simplicity, in the following, we call a biological digital twin just a digital twin because our focus is on problems in medicine and health and not in engineering.

**Figure 1:**
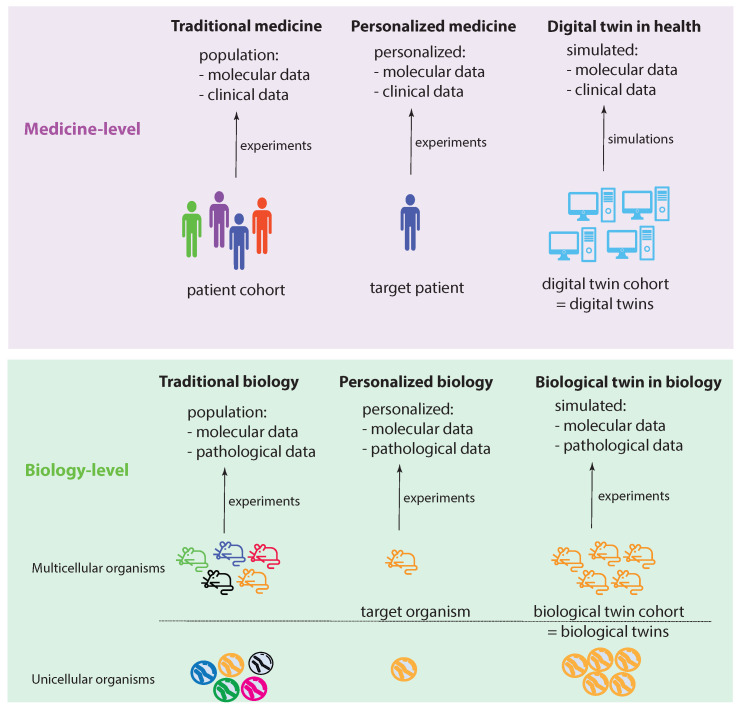
Visualizing the idea of a digital twin by comparing experimental settings in biology and medicine.

Figure 1 shows two levels: a medicine level and a biology level. These levels allow us to assume a data-centric view to discuss key concepts. We start by discussing the biology level, which shows how traditional experiments in biology are conducted. Specifically, traditional biology experiments take a representative sample from a population and conduct experiments on the members of such a sample. In the case of a multicellular organism, a sample consists of a number of animals (e.g., mice), whereas, for an unicellular organism, the sample consists of a collection of cells (e.g., S. pombe). While a sample should be representative of a population, its members are not identical in all aspects. Instead, they can be distinguished, e.g., based on their DNA. In Figure 1, this important aspect is highlighted by the different colors of the organisms.

Let us assume that we care about one member of such a sample, which can be one multicellular organism or one unicellular organism. In the former case, this would be one animal and, in the latter, one cell. We call this organism the target organism. Then, we could perform personalized biology experiments, analogous to personalized medicine experiments, by focusing on the target organism. Since we care about this individual organism, ideally, we would like to perform experiments on a sample of identical organisms to gather more experimental information. Theoretically, such identical organisms could be realized by cloning. This will allow us to create a cohort of biological twins of our target organism, whereas each biological twin is identical to the target organism.

Regarding the medicine level, the concepts of traditional biology and personalized biology can be directly extended to the medicine level by a one-to-one copy of the idea from the biology level. This leads to traditional medicine and personalized medicine (first two columns in Figure 1). However, converting the idea of a biological twin to the medicine level is, for a variety of reasons (e.g., ethical, practical), not feasible. For this reason, one needs a surrogate solution. Such a surrogate solution is provided by a digital twin. The idea of a digital twin is to use computer simulations or computer models to mimic a biological twin as closely as possible. However, due to current limitations in the understanding of biological organisms, especially of humans, this is imperfect. In Figure 1, this imperfection is highlighted by the different shades of blue for the digital twins compared to a target patient.

**Definition** **1.** *A digital twin is a computer simulation that allows us to generate biologically realistic data of a target patient.*

A key element of this definition is that a digital twin generates (biologically realistic) data. This implies that a digital twin is not a means to analyze data or, more generally, to answer questions. Instead, a digital twin is merely a surrogate for a target patient to generate data *as if* they were generated from the target patient themselves. This is important to emphasize because there is a clear distinction between data and, e.g., a machine learning method for analyzing the data. Hence, a digital twin is a computer simulation for generating data mimicking virtual biological/biomedical experiments.

In order to obtain a realistic characterization of a patient, many computer simulations need to be run to model different conditions. For instance, the administration of drugs or the effect of surgical procedures can be modeled over time to provide information about hypothetical molecular, cellular and clinical states. In Table 1, we show an overview of different interventions that can be simulated with a digital twin. Due to the fact that these are based on computer simulations and not real experiments, even the effect toward gene knockdowns can be simulated. Overall, this leads to a digital twin cohort where an individual digital twin corresponds to a particular experimental condition.

**Definition** **2.** *A digital twin cohort is a collection of digital twins each corresponding to a particular computer simulation that allows us to generate biologically realistic data of a target patient for a specific condition.*

## 3. What Advantages Do the Digital Twins Provide?

**Figure 2:**
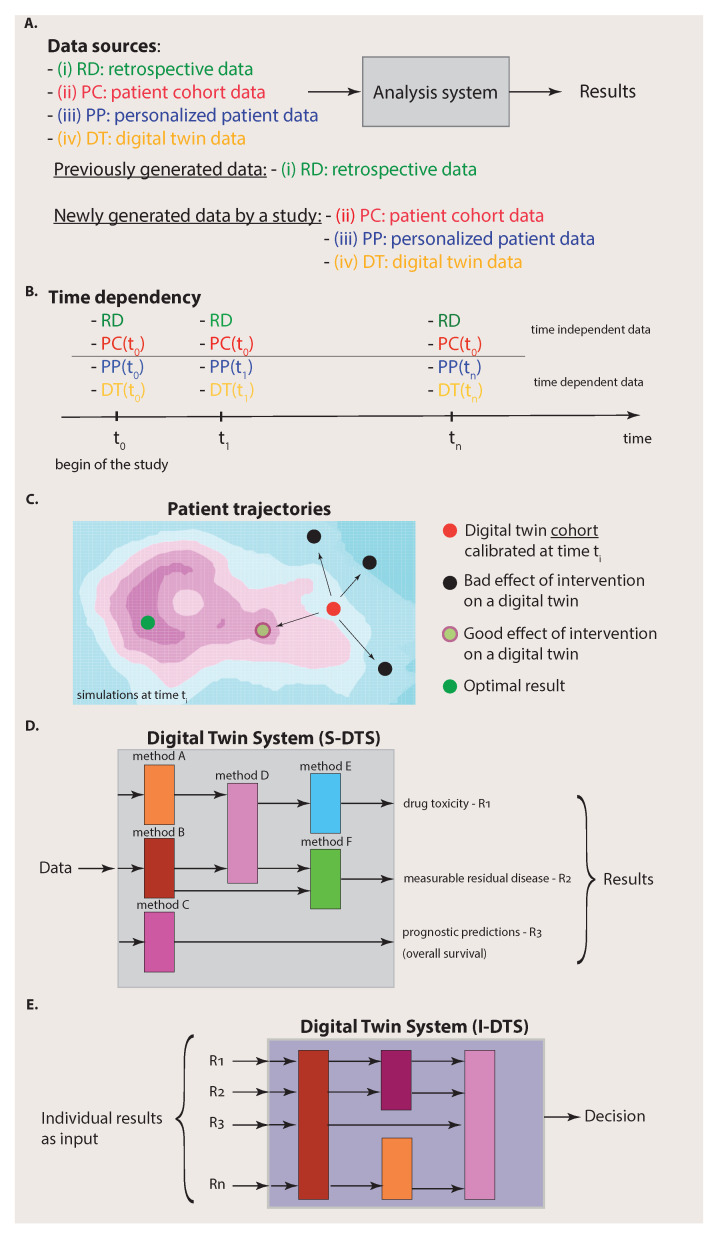
Complexity of the data (**A**–**C**) and the analysis system (**D**,**E**). (**A**): A simplified view on an analysis system that has access to four different data sources. If all four data sources (i) to (iv) are available, we call the analysis system a Digital Twin System. (**B**): Availability of data to the Digital Twin System over time. The time dependency of the different data sources is important. (**C**): Starting from a calibrated digital twin cohort at time $t_{i}$, different outcomes of various interventions are shown corresponding to different patient trajectories. (**D**): Part of the Digital Twin System for single analyses (S-DTS). (**E**): Part of the Digital Twin System for integration of analysis results (I-DTS).

From the above discussion, we have seen that digital twins generate (biologically realistic) target patient data for specific conditions. This means that digital twins establish a new data source. The benefit from this is visualized in Figure 2. Specifically, Figure 2A emphasizes that we can distinguish four different data sources from each other that are available for an analysis system: (i) retrospective data, (ii) patient cohort data, (iii) personalized patient data and (iv) digital twin data. Here, we used the term analysis system to indicate that there is more than one analysis (based on one method) that can be performed by using the four data sources. We will elaborate more on this important point below.

Importantly, before we proceed, we would like to clarify that the four data sources have a heterogeneous meaning corresponding to a categorization. Specifically, we can distinguish data that have already been generated by previous experiments from data that are newly generated by a study. Usually, the former data are deposited into a (public) database and, for this reason, we summarized these data by the term retrospective data (RD) because they have already been generated. In contrast, the data sources that correspond to patient cohort data (PC), personalized patient data (PP) and digital twin data (DT) are obtained from the current study under investigation. Hence, the data sources can be substructured according to the origin of the data, i.e., from previous experiments or the current experiments.

The connection to the previous section becomes apparent if we identify the data from data sources (ii) to (iv) with the situations visualized in the columns one to three in Figure 1, whereas the retrospective data represent data from past experiments that can be found in repositories, e.g., TCGA or LINCS [29,30]. By selecting different combinations of the data sources (i) to (iv), we can identify known special cases. For instance, for an analysis based on traditional medicine, one would only have access to data sources RD and PC whereas, for personalized medicine, this would be data sources RD, PC and PP.

In contrast, when, in addition to data sources (i) to (iii), digital twin (DT) data are also available, an analysis system can utilize data from four different data sources. Assuming that the digital twin data are of high quality, which means that they provide biologically realistic simulations and are ideally indistinguishable from biological twins, then it is clear that such an analysis system can make better predictions about the target patient because the collection of data (data sources (i) to (iv)) is more informative than the collection of any subset thereof as used, e.g., by traditional medicine (data sources RD and PC) or personalized medicine (data sources RD, PC and PP).

In order to distinguish the simulation method for generating the digital twin data from an analysis system for its analysis, we introduce the following definition.

**Definition** **3.** *An analysis system based on data sources (i) to (iv) is called a Digital Twin System.*

This definition means that a Digital Twin System has four different data sources available to perform its analyses. We would like to emphasize that this can be used for a multitude of different analyses and not just a single one, each addressing one patient-specific question (see Section 4). Hence, a Digital Twin System has a substructure depending on the patient-specific questions one wants to address.

From this discussion, we see that the advantage of digital twins is to provide a new data source that can be used in combination with other data sources ((i) to (iii)) in order to perform advanced analyses, e.g., compared to traditional medicine or personalized medicine.

It is important to note that digital twin data are structured data. This is reflected in their time dependency and their dependence on interventions. Specifically, in Figure 2B, we show the time dependency of the four data sources. One can assume that the RD data do not change over time provided that the duration of the study given by $t_{n}-t_{0}$ is short so that the information provided by databases is not significantly changed/updated during this period. In addition, the patient cohort (PC) data do not change over the duration of the study. Instead, they are usually generated at the beginning of the study (indicated by $t_{0}$). Hence, the data sources (i) and (ii) can be assumed to be static over the time of the study. This does not mean that RD data or PC data cannot contain time series of longitudinal data. It just means that their data content does not change during the study.

In contrast, personalized patient (PP) data are continuously measured for the target patient indicated in Figure 2B by a time dependency $t_{i}$, where $t_{i}$ is the point at which a measurement occurs. In addition, digital twin (DT) data do change over time because the simulations can consider updated patient information, given by $PP(t_{i})$, to modify the simulations correspondingly. Overall, this implies that the Digital Twin System provides continuous analyses and predictions over time.

A second structure of digital twin data is provided by their dependence on interventions. This can be described by the following definition.

**Definition** **4.** *A digital twin cohort is calibrated to a target patient at time $t_{i}$ and each digital twin simulates the consequences of one specific intervention.*

This means that, at a certain time $t_{i}$, a digital twin cohort is calibrated to a target patient (red point in Figure 2C) in a way that each digital twin provides the best approximation possible for simulating biologically realistic data. Then, various interventions, e.g., the administration of drugs, surgery, etc., are simulated, each with a dedicated digital twin. This leads to different outcomes corresponding to patient trajectories as a consequence of the interventions (see Figure 2C). Most of such outcomes may lead to a worsening of the health state of the patient (black points in Figure 2C), but others lead to an improvement (light green point in Figure 2C). While the optimal result corresponding, e.g., to the cure of the patient (dark green point in Figure 2C), may not be achievable by any intervention, these simulations can point to the most beneficial treatment option at a given time.

## 4. What Is a Digital Twin System?

In the previous section, we have seen that there are four different data sources available to the Digital Twin System, whereas each data source has its own substructure given by the time and intervention dependency. Such complex data also imply that the Digital Twin System needs a substructure that is a collection of different methods (see Figure 2D). Each method will allow us to make dedicated predictions about a particular patient-specific aspect. Importantly, the methods for dedicated problems can come from different fields of data science, e.g., machine learning, artificial intelligence or statistics [31,32], focusing on different aspects of data and employing different methodologies. Hence, it would not be appropriate to call such an analysis system, e.g., an AI system, because it can also comprise non-AI methods. As an example for an important non-AI method, we would like to mention survival analysis [33], which can be used for prognostic predictions, e.g., about the administration of drugs or the effect of a surgery or chemotherapy.

**Definition** **5.** *A Digital Twin System is a collection of different methods from data science, each allowing us to make dedicated predictions about a particular aspect of the target patient.*

**Figure 3:**
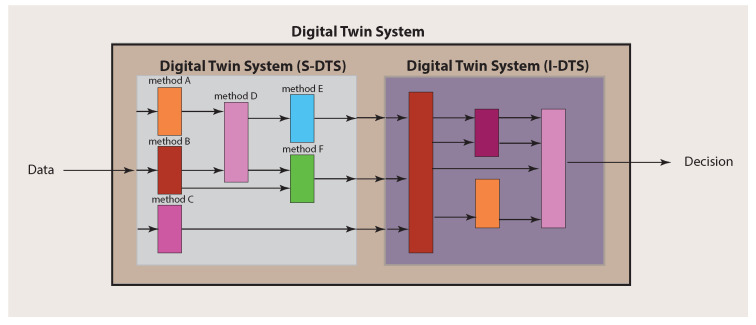
Main structure of a Digital Twin System consisting of S-DTS and I-DTS, which have themselves a complex substructure.

Importantly, for making a final decision about a patient, an integration over the single analyses results needs to be performed. In order to explicate this, we call the part of a Digital Twin System conducting the single analyses S-DTS, and the integrative part I-DTS (see Figure 3). This should clarify that a Digital Twin System is not a solitary method but a collection of different methods that can comprise techniques from a multitude of different fields, including machine learning, artificial intelligence and statistics.

## 5. Experimental Design

From the above discussion, it should be made clear that a ‘digital twin’ is neither simple nor monolithic but a complex interconnected system. However, clarifying all of its components in detail allows us to now address design aspects of experiments. Since any statistical analysis crucially depends on the underlying data, we assumed a data-centric view from the beginning of our discussion. This allowed for a clear exposition of the four data sources (see Figure 2A,B) and their characteristics and differences.

Since a Digital Twin System consists of two main parts (see Figure 3), there are also two main components to be considered for the experimental design. The first one addresses the single analysis system (S-DTS) of a Digital Twin System, which focuses on single methods for obtaining results for particular problems, whereas the second addresses the combination of the individual results performed in the integrative analysis system (I-DTS).

In Figure 2D, we depicted three potential results of an analysis corresponding to drug toxicity, measurable residual disease and prognostic predictions. Each such outcome is the result of a dedicated analysis that is typically not the same for other outcomes. Those problems have been studied extensively in the literature and, for each ‘conventional’ analysis approach, experimental design protocols have been developed [34]. Hence, for the single analyses as part of S-DTS, no new protocols are needed, but existing ones can be utilized.

The second aspect is different because it requires the combination of all individual results obtained in the previous step corresponding to the integrative analysis part (I-DTS) of a Digital Twin System (see Figure 2E and Figure 3). This combination could either form a meta-analysis or a Bayesian approach [35]. Potentially, this step is more challenging than the previous one, especially when combining heterogeneous results, e.g., when not all outcomes are *p*-values. Either way, the amalgamated results will allow for personalized, comprehensive decision making about the patient by considering all individual data sources and intermediate results.

## 6. Applications

In order to provide an outlook on the wider implications of a Digital Twin System, we outline in the following potential applications in research and the clinics.

With respect to the artificial pancreas model [17] for type I diabetes discussed above, we think that it is fair to say that, currently, a full multiscale-modeling approach over a multitude of levels is very hard to reach. Nevertheless, there are a number of feasible applications that can be studied. The first one is focused on gene expression. While a biological cell comprises intricate regulatory programs between the DNA, mRNA and protein level, a realistic simulation of all aspects thereof is currently too demanding. For this reason, focused models have been developed that provide simulations of the gene expression. Specifically, mathematical models of gene expression utilize a variety of different approaches, whereas the most prominent ones are based on thermodynamic models, differential equation models and Boolean models [36,37,38]. Such models can provide a digital twin for gene expression, which is an informative level for the dynamic activities of genes and proteins. Furthermore, gene expression also captures the effects of perturbations, e.g., by the administration of drugs. However, as a note, we would like to add that the most realistic models developed in this context so far are for prokaryote cells, e.g., [39,40,41].

Another application is for disorders that are, so far, well-studied, providing ample data. Such data are needed for designing simulation models enabling *semi-mechanistic* models. This means that, instead of relying on validated mechanistic models, data are used to leverage an approximate model that is functional within a well-restricted molecular domain but does not fully incorporate all mechanistic details. For instance, cancer in general is among the most widely studied disorder families, whereas breast cancer and lung cancer are prominent subtypes thereof. For each of those cancer types, tens of thousands of genomics data samples are available from public repositories, e.g., from TCGA or GEO [29,42]. This can facilitate the development of approximate models for dedicated disease subcategories that can function as a proxy digital twin.

Finally, we think that Mendelian diseases, e.g., cystic fibrosis, sickle cell anaemia or muscular dystrophy, would form excellent testbeds because much of the complexity of non-Mendelian disorders is omitted. Specifically, while complex disorders employ gene products on a systems scale that are connected via large gene regulatory networks, Mendelian diseases may be limited to smaller circuits. Hence, the mathematical modeling techniques could be more easily deployed for Mendelian diseases that exhibit a reduced dynamic behavior without being trivial.

## 7. Ethical Considerations

The above discussion shows that the concept of a digital twin leads to a new data source that corresponds to simulated data. From an ethical point of view, this raises several concerns that we address in the following. We would like to emphasize that, in a health context, such a discussion is imperative because of the involvement of patients. This is potentially different from engineering applications of a digital twin, which deal exclusively with an inanimate nature.

The first issue relates to the nature of the data themselves. Specifically, in science, it is common to introduce novel measurement devices or technologies once in a while. A fairly recent example from genomics is given by the next-generation-sequencing (NGS) technology [43,44]. In general, high-throughput devices based on NGS or other novel technologies are capable of measuring either new aspects of an experiment or providing a more refined approach to previous problems. However, regardless of those details, such measurement devices *always* provide experimental data. In contrast, a digital twin *always* provides simulated data. This is an important qualitative difference.

In order to make this qualitative difference clear, let us consider two examples. In the first example, the worst consequence of a false treatment decision may result in a discomfort of the patient, whereas, in a second example, a false treatment decision may lead to death. Certainly, both cases can occur in any data analysis. However, when using simulated data for such a decision, the case of a failure (especially for example two) is difficult to accept because of the indirect nature of such data. Hence, extensive testing, clinical validation and certification are required before approaches based on a digital twin can reach the clinic or any practical application in medicine or health.

The second issue is regarding the explainability of a Digital Twin System. Currently, explainable artificial intelligence (AI) [45,46,47,48] is under intense investigation because, on the one hand, e.g., deep learning neural networks are lacking usually an easy and insightful interpretation, and, on the other hand, many application domains require explainable models and shy away from *black-box* prediction models (as deep neural networks), even when they are state-of-the art. The latter is especially true for medicine and health-related areas [49]. For a Digital Twin System, the situation is even more complex because, as discussed above (see Definition 5), a Digital Twin System is not a monolithic approach but a collection of different methods from data science. Hence, establishing an *explainable Digital Twin System* requires that all methods that it combines are explainable themselves. This is very demanding and challenging.

Interestingly, there is one further problem in this context which is given by a digital twin itself. Since a digital twin is a simulation model, also the method for simulating the data, and not only for analysing the data, needs to be explainable. Given the probabilistic nature of such simulation methods [50,51], this is unlikely to be less demanding.

At the moment, it is unclear if *explainability* is a reachable goal for conventional AI methods [52], and it remains to be seen how this could be expanded to even more complex analysis systems, such as a Digital Twin System. Hence, aside from methodological questions, there are severe and unprecedented ethical issues that need to be addressed in order to use a digital twin in medicine or health.

## 8. Discussion

In recent years, several publications appeared that provided a definition of a digital twin in various application domains. For instance, in [53], the authors gave a comprehensive survey about many aspects of a digital twin, including its definition. Specifically, in total, 31 articles were cited that provide a definition of a digital twin. However, upon inspection, none of these 31 articles provide a data-centric machine learning perspective, but instead provide an engineering-based characterization. While useful in certain contexts, this is not sufficient if the goal is the analysis of data. For this reason, we re-visited the definition of a digital twin from a different perspective. We centered this perspective around the meaning of a digital twin, which is the generation of (simulated) data. Hence, our perspective is data-centric.

As a result, from our discussion, we find the reason for why previous definitions of a digital twin appear cluttered, unprecise and convoluted. The reason is an overloading of the term ‘digital twin’ and the neglect of introducing defined substructures. In contrast, from our analysis, we find the following main substructures, for which, we introduced definitions:

- A digital twin simulates data.
- A digital twin cohort is a collection of digital twins.
- There are four types of data sources, and digital twin data are one of these.
- Digital twin data are time-dependent.
- A digital twin cohort is calibrated to a target patient at time $t_{i}$.
- A Digital Twin System consists of two main parts (S-DTS and I-DTS), which are collections of analysis methods.

In summary, this means that a digital twin is like a Matryoshka doll (Russian dolls), where one doll is stacked into another one. The current literature seems to overlook these substructures, which lead to discussions about ‘digital twins’ (dolls) without precise differentiations and connections. Instead, we gave the various ‘dolls’ different names and defined their connections.

## 9. Conclusions

In this paper, we provide a discussion about the concept of a digital twin. Our analysis reveals that the term ‘digital twin’ as used in the literature is like a Matryoshka doll. For this reason, we unstacked the concept via a data-centric machine learning perspective, allowing us to define its main components. As a result, we found six components that, together, define the concept of a ‘digital twin’. As a consequence, we suggest to use the term Digital Twin System instead of digital twin because this implies the following.

1. A Digital Twin System is a complex entity with interconnected substructures.
2. Each substructure needs to be optimized for a given problem setting, e.g., in medicine or health.
3. A digital twin is just one method for simulating intervention-dependent data.

It is unquestionable that a Digital Twin System holds great promise for applications in medicine and health. However, there are great challenges ahead, methodologically as well as ethically, which need to be overcome first before it can have an impact on personalized and precision medicine. Given the complex nature of a Digital Twin System, this will not be an easy task.

## Figures and Tables

**Table 1:** An overview of different intervention types that can be simulated by different digital twins.

Intervention Type	External Condition	Internal Condition
	environmental changes	knockdown effects
	diet changes	gene therapy
	surgery	pharmaceutical interventions

## Data Availability

Not applicable.
